# Incidence and Prevalence of Cancer in Colombia: The Methodology Used Matters

**DOI:** 10.1200/JGO.17.00008

**Published:** 2017-07-06

**Authors:** Omaira Valencia, Gilberto Lopes, Patricia Sánchez, Lizbeth Acuña, Daniel Uribe, Jaime González

**Affiliations:** **Omaira Valencia**, **Patricia Sánchez**, **Lizbeth Acuña**, and **Daniel Uribe**, Cuenta de Alto Costo, Fondo Colombiano de Enfermedades de Alto Costo; **Jaime González**, Asociación Colombiana de Hematologíay Oncológica, Bogotá, DC, Colombia; and **Gilberto Lopes**, University of Miami Sylvester Comprehensive Cancer Center, Miami, FL.

## Abstract

**Purpose:**

Incidence and prevalence are important factors in policy making and planning in health care systems. The aim of this study was to compare two different estimates of the incidence and prevalence of cancer in Colombia—real-world data from the health care system and estimates from cancer registries.

**Materials and Methods:**

Data from all providers were aggregated by the High-Cost Diseases Office (Cuenta de Alto Costo [CAC]). The real-world, age-standardized observed incidence (OI) and observed prevalence (OP) rates were calculated using the number of patients with a diagnosis of cancer who were cared for in the national health system between 2014 and 2015. The registry estimated incidence (EI) and estimated prevalence (EP) were extracted from GLOBOCAN population fact sheets for 2012, which use data from four Colombian city-based registries and extrapolate survival using the average for Asian countries, together with registries from Uganda and Zimbabwe.

**Results:**

A total of 130,441 patients were analyzed. The OI of cancer in Colombia was 69.2 and the OP was 479 (per 100,000 people) in early 2015, whereas the EI was 175.2 and the 5-year EP was 501.2 (per 100,000 people), showing a higher estimate from GLOBOCAN data for 2012 than was observed in early 2015 by the CAC. Some differences were higher in specific cancers.

**Conclusion:**

Because of differences in methodology, the EI and the EP are not comparable to the OI and the OP. Policymakers need robust and current information to prioritize disease prevention and control programs. In Colombia, the OI and the OP—calculated by the CAC with data from the whole country—offer an opportunity for a more precise real-world estimation of patients with cancer in Colombia.

## INTRODUCTION

In 2012, there were 14.1 million new cancer cases worldwide, 8.2 million deaths as a result of cancer, and 32.6 million people still living who had had a diagnosis of cancer for > 5 years. Almost one half of these cases and approximately 60% of all deaths came from low- and middle-income countries.^1^ In the same year, the mortality associated with lung malignancies alone (including those of the trachea and bronchus) was the fifth cause of death in the world, after cardiovascular diseases and upper and lower respiratory tract infections.^2^

In Colombia, the National Statistics Department reported that between 2014 and 2015, lung and stomach neoplasms were two of the 10 main causes of death. The most common malignancies in the country were breast and cervical cancer for women, prostate cancer for men, and leukemia for children.^3^

Moreover, as the costs of diagnosing and treating cancer rapidly rise, stakeholders worry that with the expected increase in the burden of disease, economic and social expenditures will become untenable, especially as they pertain to new drugs and technologies.^3-5^

The Colombian health system is a public-private system that guarantees high coverage of the national population. The system is regulated by the national government through the Ministry of Health, is monitored by the National Superintendent of Health, and covers 94.6% of Columbian citizens.^6^ Private insurance companies called health promoting enterprises (Empresas Promotoras de Salud [EPSs]) manage the health care of their specific insured populations.

A group of EPSs manages the health of the whole population of workers; premium payments come from mandatory contributions by employees and employers. Another group of EPSs manages the health of the poor nonworking population, and their payments come from contributions from working citizens (which are regulated and mandatory) and from the government. Colombia updated its mandatory health plan in 2011 and incorporated an important number of new technologies for cancer control.^6^ Because of the importance of having adequate information for decision making in the health care system and to guide the regulatory process, the National Administrative Cancer Registry (NACR) was created in 2012 to cover all of the national territory. Health insurers and providers are mandated to report data on all patients with cancer to the High-Cost Diseases Office (Cuenta de Alto Costo [CAC]).^7^

Incidence and prevalence, which indicate the burden of disease, are important factors in policy making and planning in health care systems. The aim of this study was to compare two different estimates of the incidence and prevalence of cancer in Colombia—real-world data from health care insurers and providing institutions, and estimates from cancer registry information.

## MATERIALS AND METHODS

### Observed Incidence and Prevalence in the NACR

Data from all EPSs were compiled by the CAC into the NACR. Real-world data were extracted from the administrative registry; age-standardized observed incidence (OI) and observed prevalence (OP) rates were calculated for adult patients living with a diagnosis of cancer who were reported to the CAC between January 2, 2014, and January 1, 2015, from Colombia’s 32 departments; diagnosis could have been received in this period or earlier.

To ensure the quality of the data input and decrease, as much as possible, the risk of inaccurate information being entered into the database, CAC audited all information reported from EPSs throughout the country and verified clinical information. Auditors were trained by oncologists and supervised by public health specialists with training and experience in auditing.

The number of cancer cases was computed for each geographic location and by sex. Furthermore, the entire Colombian population older than 15 years of age was used as the denominator. Estimates include all cancer types and exclude nonmelanoma skin cancers.

For specific cancer types, codes were used from the International Classification of Diseases, Tenth Revision as follows: (1) hematologic malignancies: Hodgkin lymphoma (C81), non-Hodgkin lymphoma (C82 to C85, C96), multiple myeloma (C88, C90), and leukemia (C91 to C95); and (2) solid tumors: lip and oral cavity (C00 to C08); nasopharynx (C11); other pharynx (C09 to C10, C12 to C14); esophagus (C15); stomach (C16); colorectum (C18 to C21); liver (C22); gallbladder (C23 to C24); pancreas (C25); larynx (C32); trachea, bronchus, and lung (C33 to C34); melanoma of the skin (C43); Kaposi’s sarcoma (C46); breast (C50); cervix uteri (C53); corpus uteri (C54); ovary (C56); prostate (C61); testis (C62); kidney (C64 to C66); bladder (C67); brain, nervous system (C70 to C72); and thyroid (C73).

Incidence was calculated as the number of patients who were diagnosed in the reported period, taking the date of the pathology report as the moment of disease diagnosis. Prevalence was determined to be living patients who had been diagnosed at any time. STATA V13 software (STATA, College Station, TX) was used for statistical analysis.

### Estimated Incidence and Estimated Prevalence From GLOBOCAN

The registry estimated incidence (EI) and the estimated prevalence (EP) were extracted from GLOBOCAN population fact sheets for 2012. The four cancer registries, which cover < 10% of Colombia, are from Cali, Bucaramanga, Manizales, and Pasto, and covered 8% of the population between 2003 and 2007. National mortality rates (2000 to 2009) were projected to 2012 and applied to the 2012 population.^8^

GLOBOCAN projected data collected between 2003 and 2007 to 2012 and used recorded information in country-specific registries to calculate cancer incidence. Prevalence was determined from incidence estimates and the regional average of observed survival by cancer and age group.^9^

For South American countries, the sources of survival used in the estimates of cancer prevalence were determined from an unweighted average of survival rates from registries in South Korea, Singapore, China, India, Thailand, Uganda, and Zimbabwe.^8^

### Difference Ratio Between CAC Database and GLOBOCAN

We calculated the difference ratio (DR) between the GLOBOCAN and the NACR to determine how many times the EP or the EI was above or below the OI or the OP.

## RESULTS

A total of 178,879 records were retrieved. To make the results comparable to GLOBOCAN data, the analysis was restricted to the 75% of patients ≥15 years of age (130,441 cases).

### OI

There were 30,675 new cases reported in a 12-month period between 2014 and 2015 in the Colombian health care system. Of these, 21,994 were eligible for analysis on the basis of their age and specific code from the International Classification of Diseases, Tenth Revision; nonmelanoma skin cancer was excluded. Of the 8,681 excluded patients, 4,354 were diagnosed with other skin malignances (50.1%), 1.11% had secondary tumors, and the remainder had either cancer of other mesothelial tissues or other badly classified tumors. Noninvasive tumors were excluded from this analysis to make the data comparable to the GLOBOCAN data.

The six most common malignancies were breast (17.2), prostate (8.7), cervical (6.4), thyroid (4.9), stomach (3.2), and ovary (3.2) cancers (Table 1).

**Table 1:**
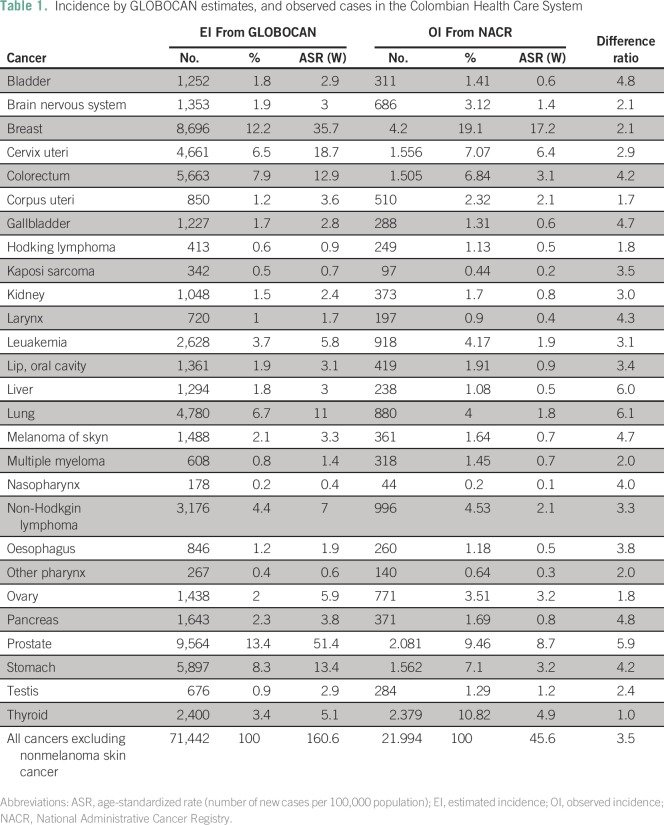
Incidence by GLOBOCAN estimates, and observed cases in the Colombian Health Care System

### EI

GLOBOCAN estimated a total of 71,442 new cases of cancer in Colombia in 2012, with an incidence of 160.6 new cases per 100,000 population. The most common malignancies were prostate (51.4), breast (35.7), cervical (18.7), stomach (13.4), colorectal (12.9), and lung cancers (11).

The widest gaps (highest DR) between incidences were for lung cancer (6.1), followed by liver cancer (6.0) and prostate cancer (5.9). The lowest DRs were for thyroid (1.0), followed by Hodgkin lymphoma (1.8) and corpus uteri cancer (1.7; Table 1).

### EP and OP

The 5-year EP was 501.2 per 100,000 people. The number of prevalent cases of cancer in 2012 estimated by GLOBOCAN is higher than the OP in early 2015 calculated by the CAC’s assessment of the NACR (369.4 per 100,000). However, for breast, thyroid, and non-Hodgkin lymphoma, the OP was higher than the EP.

Using GLOBOCAN data, the most prevalent malignancies were breast (177.6), prostate (169.3), cervix uteri (85.6), colorectal (41.2), stomach (28.8), and thyroid (26.7). Conversely, using the CAC data, the six most common malignancies were breast (201.9), prostate (97.7), cervix uteri (51.0), thyroid (31.8), colorectal (20.6), and non-Hodgkin lymphoma (18.8; Table 2).

**Table 2:**
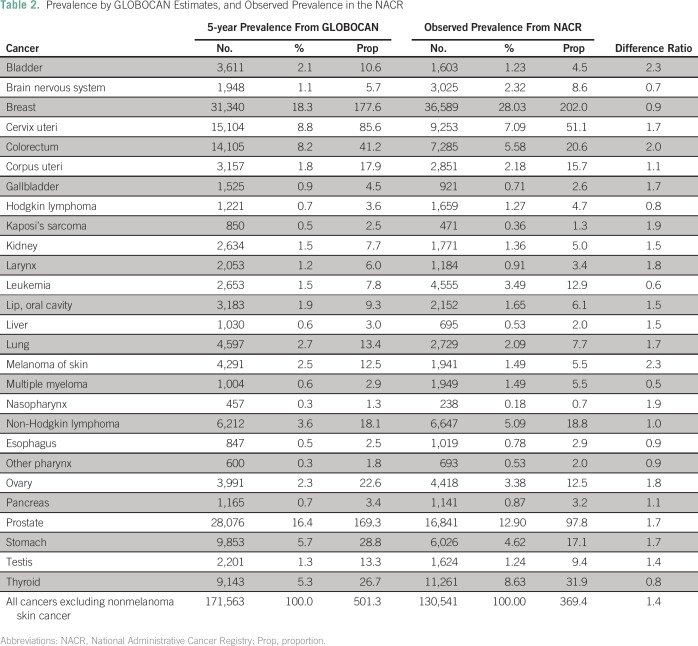
Prevalence by GLOBOCAN Estimates, and Observed Prevalence in the NACR

In certain cancers, specifically those of the brain and breast, Hodgkin lymphoma, leukemia, multiple myeloma, cancer of the esophagus and other pharynx and thyroid, there was a constant between the EP and OP, and the DR was < 1. Conversely, for neoplasms of the bladder and colorectum and melanoma of skin, the DR was > 2 (Table 2).

## DISCUSSION

In this article, we show that, because of different methodologies, there are significant differences in the EI and the EP and the OI and the OP of cancer between GLOBOCAN and the Colombian NACR data. There are several potential explanations for these differences. The 5-year EP in GLOBOCAN is calculated on the basis of incidence and survival rates. Data on incidence come from four Colombian city-based registries that have been classified as high quality. Survival for South American countries in GLOBOCAN was extrapolated using the average for Asian countries, together with registries from Uganda and Zimbabwe.

The OI and the OP come from information collected and audited by the CAC. Under law 234 of 2014, the Ministry of Health mandated the reporting of cases of cancer to the CAC from all EPSs including public, private, and mixed, and from municipality health secretariats.^10^

Moreover, GLOBOCAN calculated the EP using mortality and survival rates available in 2007, and projected to 2012,^8,11^ whereas we determined the OP by the number of cases observed during the period reported.

A few malignancies had a higher OP in the NACR than in the GLOBOCAN estimate: breast, thyroid, and non-Hodgkin lymphoma. For breast cancer, these differences may reflect the development of early detection programs and increased actual versus expected survival^12^; for non-Hodgkin lymphoma, the difference could be explained by the multiracial diversity seen in the country.^13^ It has been documented that in Colombia, 37% of the population is white and 10.6% is of African descent^14^; both ethnicities have important risk factors related to the development of non-Hodgkin lymphoma.^15^ In Colombia, 14.4% of the population recognize themselves as native or of African descent.^16^ Moreover, an increase in the use of ultrasound because of better access to the health care system may explain the higher observed rates for thyroid cancer, as has been seen in other countries, such as South Korea.^17,18^ For all other malignancies, the EP was higher than the OP. The EI was higher than the OI for all disease sites.

Under-reporting may account for these lower numbers, although it is mandatory by law to provide information to the CAC. All EPSs must report cases of cancer or they do not receive the corresponding payments. Although that does not eliminate the risk of under-reporting, it certainly decreases its likelihood. It is possible, however, that cases of nondiagnosed malignancies with high mortality are missed.

Nearly all Colombians are covered by the health care system (< 6% of the population is not insured). Differences between the reference estimates extrapolated by GLOBOCAN and the city registries in Colombia, and differences in the estimates of the overall population of the country are the most probable causes of the discrepancies we described. The methodologies may actually be complementary. GLOBOCAN may have overestimated data related to poor prevention and early detection programs by using the paradigm of low-income countries^19^; this gap could also be explained by under-reporting to the administrative cancer registry. Moreover, Bogotá (the capital city, which has 11% of the total population) is not represented in the GLOBOCAN estimate.National policymakers need robust and current estimates of cancer incidence and prevalence to develop policies to create and improve high-quality services.^20,21^ The OI and the OP obtained from the CAC through the NACR, with data from all the 32 departments in the country, offer an opportunity for a more precise real-world estimation of new cases each year and of the number of patients living with cancer. In the future, with more information on outcomes, incidence, and prevalence, data from the NACR may also allow comparison of outcomes by each health care insurer and provider.^22^ This initial report from 2015 is a starting point that needs to be built on with the participation of all stakeholders, including patients, health care providers, insurers, and policymakers, to ensure the continuous development of high-quality cancer services in Colombia.
